# Potassium (1*R*,4*R*,5*S*,8*S*)-4,5,8-trihy­droxy-3-oxo-2,6-dioxabicyclo­[3.3.0]octane-4-sulfonate dihydrate

**DOI:** 10.1107/S1600536812048672

**Published:** 2012-12-05

**Authors:** Alan H. Haines, David L. Hughes

**Affiliations:** a School of Chemistry, University of East Anglia, Norwich NR4 7TJ, England

## Abstract

The title salt, K^+^·C_6_H_7_O_9_S^−^·2H_2_O, formed by reaction of dehydro-l-ascorbic acid with potassium hydrogen sulfite in water, crystallizes as colourless plates. The potassium ion is coordinated by eight O atoms arising from hy­droxy or sulfonate groups. The sulfonate group is bonded to the C atom neighbouring that of the lactone carbonyl group. As is commonly observed in crystalline l-ascorbic acid derivatives, the O atom of the primary hy­droxy group is linked to the second C atom from the lactone C atom, forming a hemi-acetal function, thereby creating a bicyclic system of two fused five-membered rings, both of which have envelope conformations with one of the shared C atoms as the flap. Addition of the sulfur nucleophile occurs from the less hindered face. One of the two independent lattice water mol­ecules has hydrogen bonds to sulfonate O atoms of two different anions and is the acceptor of bonds from hy­droxy groups of two further anions; the second lattice water mol­ecule donates to the carbonyl and a hy­droxy O atom in different anions, and accepts from a hy­droxy O atom in a further anion. Thus, through K—O coordination and hydrogen bonds, the potassium cations, sulfonate anions and water mol­ecules are linked in a three-dimensional network.

## Related literature

For the first synthesis of the title compound, see: Ingles (1961 ▶). For related studies on crystalline properties of hydrogen sulfite addition products of carbohydrates and their structures, see: Cole *et al.* (2001 ▶); Haines & Hughes (2010 ▶, 2012 ▶). For examples of related bicyclic structures based on dehydro-l-ascorbic acid, see: Hvoslef (1972 ▶); Yvin *et al.* (1982 ▶).
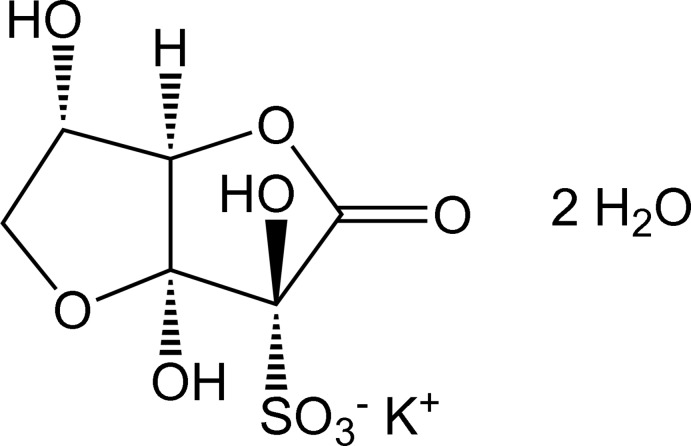


## Experimental

### 

#### Crystal data

K^+^·C_6_H_7_O_9_S^−^·2H_2_O
*M* *_r_* = 330.31
Orthorhombic, 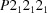

*a* = 6.21040 (15) Å
*b* = 6.93014 (16) Å
*c* = 26.7851 (7) Å
*V* = 1152.80 (5) Å^3^
*Z* = 4
Mo *K*α radiation
μ = 0.70 mm^−1^
*T* = 140 K
0.80 × 0.40 × 0.10 mm

#### Data collection

Oxford Diffraction Xcalibur Sapphire3 CCD diffractometer
Absorption correction: multi-scan (*CrysAlis RED*; Oxford Diffraction, 2010 ▶) *T*_min_ = 0.851, *T*_max_ = 1.000
15452 measured reflections
2022 independent reflections
2012 reflections with *I* > 2σ(*I*)
*R*_int_ = 0.026

#### Refinement

*R*[*F* ^2^ > 2σ(*F* ^2^)] = 0.018
*wR*(*F* ^2^) = 0.048
*S* = 1.13
2022 reflections
214 parameters
All H-atom parameters refined
Δρ_max_ = 0.22 e Å^−3^
Δρ_min_ = −0.24 e Å^−3^
Absolute structure: Flack (1983 ▶), 806 Friedel pairs
Flack parameter: 0.00 (4)

### 

Data collection: *CrysAlis CCD* (Oxford Diffraction, 2010 ▶); cell refinement: *CrysAlis CCD*; data reduction: *CrysAlis RED* (Oxford Diffraction, 2010 ▶); program(s) used to solve structure: *SHELXS97* (Sheldrick, 2008 ▶); program(s) used to refine structure: *SHELXL97* (Sheldrick, 2008 ▶); molecular graphics: *ORTEPIII* (Johnson, 1976 ▶) and *ORTEP-3 for Windows* (Farrugia, 2012 ▶); software used to prepare material for publication: *SHELXL97* and *WinGX* (Farrugia, 2012 ▶).

## Supplementary Material

Crystal structure: contains datablock(s) I, global. DOI: 10.1107/S1600536812048672/wm2701sup1.cif

Structure factors: contains datablock(s) I. DOI: 10.1107/S1600536812048672/wm2701Isup2.hkl

Supplementary material file. DOI: 10.1107/S1600536812048672/wm2701Isup3.cdx

Supplementary material file. DOI: 10.1107/S1600536812048672/wm2701Isup4.cml

Additional supplementary materials:  crystallographic information; 3D view; checkCIF report

## Figures and Tables

**Table 1:** Hydrogen-bond geometry (Å, °)

*D*—H⋯*A*	*D*—H	H⋯*A*	*D*⋯*A*	*D*—H⋯*A*
O2—H2*O*⋯O8^i^	0.77 (3)	1.91 (3)	2.6533 (18)	162 (3)
O3—H3*O*⋯O8	0.79 (3)	1.87 (3)	2.6525 (18)	171 (3)
O5—H5*O*⋯O9^ii^	0.70 (3)	2.01 (3)	2.684 (2)	162 (3)
O8—H8*OA*⋯O22^iii^	0.81 (3)	2.07 (3)	2.8141 (18)	155 (3)
O8—H8*OB*⋯O23^i^	0.78 (3)	2.13 (3)	2.7798 (18)	142 (2)
O9—H9*OA*⋯O5^iv^	0.77 (3)	2.13 (3)	2.869 (2)	161 (3)
O9—H9*OB*⋯O1	0.83 (3)	1.95 (3)	2.7852 (19)	175 (3)

Symmetry codes: (i) 
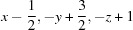
; (ii) 
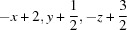
; (iii) 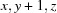; (iv) 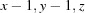.

**Fig. 1.**
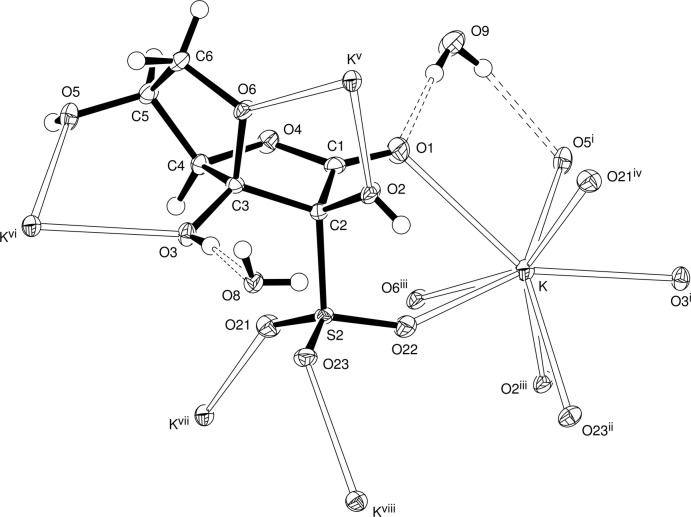
View of the expanded asymmetric unit of K+.C6H7O9S-.2H2O, showing the conformation of the anion, the coordination of the potassium cation and the locations of the water molecules; all the potassium ions bonded to the anion are included. In all the figures, thermal ellipsoids are drawn at the 50% probability level, and hydrogen atoms are drawn as spheres of arbitrary size. Superscripts in the atom labels denote symmetry operations as defined in the Tables of molecular dimensions and hydrogen bonds.

**Fig. 2.**
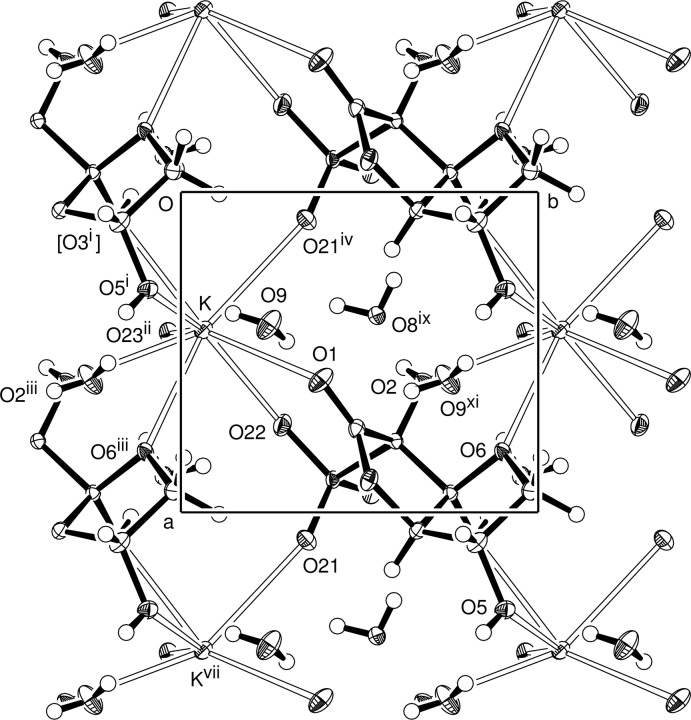
A section through the unit cell, showing the linking of the different moieties through K—O coordination bonds.

**Fig. 3.**
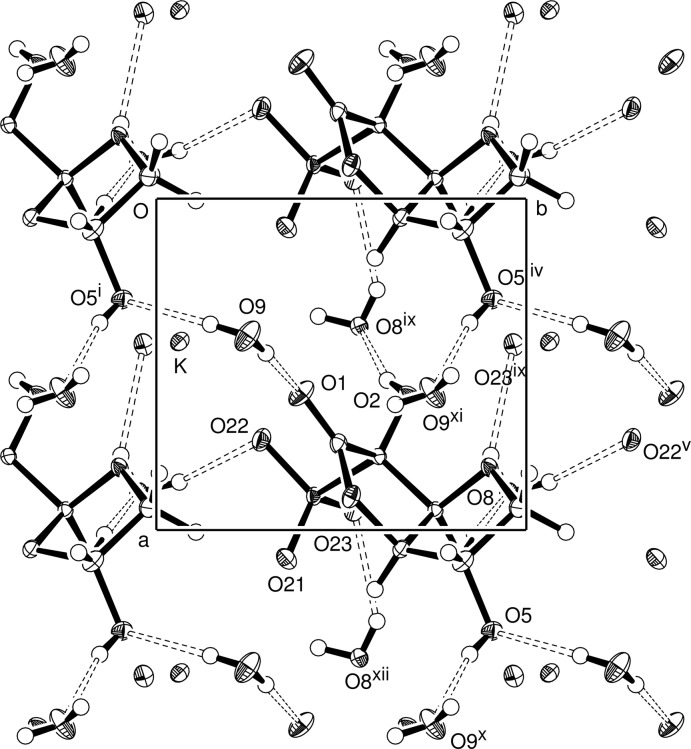
A section through the unit cell (as Figure 2), showing the linking of the different moieties through hydrogen bonds (dashed lines).

## supplementary crystallographic information

### Comment

The addition of hydrogen sulfite (bisulfite) anion to carbonyl compounds to form sulfonic acid salts has found use in the purification of aldehydes and some ketones. Certain carbohydrates, despite existing preponderately in a hemi-acetal form, also give such adducts and the crystalline structures of the potassium sulfonic acid salts of D-glucose and D-mannose (Cole *et al.*, 2001), D-galactose (Haines & Hughes, 2010) and the sodium sulfonic salt of D-glucose (Haines & Hughes, 2012) have been determined by X-ray crystallographic studies and all shown to exist in an acyclic form.

Ingles (1961) reported the preparation of an addition product between potassium hydrogen sulfite and dehydro-L-ascorbic acid. This compound, with carbonyl functionality at C1, C2 and C3, has potentially three sites of attack by the anion, but the carbonyl group at C1 is incorporated in a lactone structure and therefore is the least likely to undergo nucleophilic attack by the sulfur but the question of formation of a C2 or C3 adduct remained open. Our present study was undertaken to determine the structure of this compound.

Preparation of the adduct by reaction of potassium hydrogen sulfite (generated in aqueous solution from potassium metabisulfite) and dehydro-L-ascorbic acid in water by the published procedure gave, as reported, the adduct as a dihydrate with properties (m.p. and [α]_D_) in agreement with those described (Ingles, 1961). HRESIMS (negative ion mode, methanol solution) did not show an expected peak at m/e 254.9816 for [C_6_H_7_O_9_S]^-^ but showed peaks at 173.0089 for the dehydro-L-ascorbic acid anion (calculated for [C_6_H_5_O_6_]^-^: m/*z* 173.0092) and the ion of the methanol adduct at 205.0349 (calcd for [C_7_H_9_O_7_]^-^: m/*z* 205.0354), indicating instability of the adduct in the methanol solution.

The crystal structure indicates attachment of the sulfur at C2 on the opposite side of the fused ring formed by attack of O6 on the C3 carbonyl function (Figure 1). This bicyclic motif for L-ascorbic acid derivatives in which the two rings share the C3—C4 bond is a common feature revealed in many crystal structures determined by X-ray crystallography, for example the dehydro-L-ascorbic acid dimer (Hvoslef, 1972) and the marine natural product delesserine (Yvin *et al.*, 1982). Both rings have envelope conformations and C3 is the flap out-of-plane atom in each. The potassium ions are eight-coordinate (Figures 1 and 2) with a coordination sphere between that of a square antiprism (in which one of the square planes is O22, O23^ii^, O2^iii^, and O6^iii^; for symmetry codes, see: 'Geometric parameters Table') and dodecahedral (in which the pairs of `pseudo-equatorial' B-site atoms are O22, O5^i^ and O21^iv^, O2^iii^). Each potassium ion is bonded to O atoms of five different anions with K—O bond lengths in the range 2.6757 (13) – 3.0265 (13) Å. Of the two lattice water molecules, that containing O8 has hydrogen bonds to oxygen atoms in different sulfonate groups (Table1, Figure 3) and is the acceptor of two hydrogen bonds, from H2O and H3O in different anions, leading to an approximately tetrahedral arrangement about the O8 atom. The water molecule of O9 accepts a single hydrogen bond from an H5O atom and donates two hydrogen bonds, to O1 and O5, in different anions (Figure 3); the trigonal arrangement at the O9 atom has an umbrella shape. Thus, through K—O coordination and hydrogen bonds, potassium cations, sulfonate anions and water molecules are linked in a three-dimensional network.

### Experimental

The title compound was prepared by a procedure similar to that described (Ingles, 1961). L-Ascorbic acid (1.76 g) was oxidized by shaking with iodine (2.48 g) in MeOH (15 ml) and the solution neutralized with basic lead carbonate (7 g), then filtered through kieselguhr. The syrup obtained on evaporation of the solution was dissolved in water (1.2 ml) and added to a solution of potassium hydrogen sulfite made by dissolving potassium metabisulfite (1.11 g) in water (1.6 ml). The crystals that formed were collected, washed with 95% EtOH and dried under vacuum over P_2_O_5_, m.p. (with swelling) 401–403 K with gradual decomposition to 473 K [lit. slow decomposition above 423 K (Ingles, 1961)]; [α]_D_^24^ +38.5 (*c*, 0.74, 9:1 H_2_O:HOAc), lit. [α]_D_ +35 (*c* 1.5, 9:1 H_2_O:HOAc). ^1^H NMR (D_2_O, 400 MHz, reference *Me*_3_COH at δ_H_ 1.24): δ 4.95 (br s, H-4), 4.63 (ddd, *J*_5,6a_ = 5, *J*_5,6 b_ = 2.5, *J*_4,5_ = 0.6 Hz, H-5), 4.24 (dd, *J*_6a,6b_ = 10.4 Hz, H-6a), 4.18 (dd, H-6 b). ^13^C NMR (D_2_O, 100 MHz, referenced to *Me*_3_COH at δ_C_ 30.29): δ 172.33 (C1), 107.17 (C3), 88.88 (C2), 88.70 (C4), 76.06 (C6), 73.64 (C5).

HRESIMS (negative ion mode, measured in MeOH solution) gave no peak at the expected *m/z* [C_6_H_7_O_9_S_1_]^-^ but predominant peaks were observed at *m/z* 173.0089 ([C_6_H_5_O_6_]^-^), and 205.0349 ([C_7_H_9_O_7_]^-^) which indicated (i) decomposition of the sulfonate in solution to dehydro-L-ascorbic acid, and (ii) addition of methanol to this decomposition product.

### Refinement

Hydrogen atoms were located in difference maps and were all refined freely except, in the final cycles, the U_iso_ parameters of the methine hydrogen atoms were set at 1.2^.^U_eq_ of the parent carbon atoms.

### Crystal data

**Table:**

K^+^·C_6_H_7_O_9_S^−^·2H_2_O	*F*(000) = 680
*M**_r_* = 330.31	*D*_x_ = 1.903 Mg m^−^^3^
Orthorhombic, *P*2_1_2_1_2_1_	Mo *K*α radiation, λ = 0.71073 Å
Hall symbol: P 2ac 2ab	Cell parameters from 9940 reflections
*a* = 6.21040 (15) Å	θ = 3.6–32.6°
*b* = 6.93014 (16) Å	µ = 0.70 mm^−^^1^
*c* = 26.7851 (7) Å	*T* = 140 K
*V* = 1152.80 (5) Å^3^	Plate, colourless
*Z* = 4	0.80 × 0.40 × 0.10 mm

### Data collection

**Table:**

Oxford Diffraction Xcalibur 3/Sapphire3 CCD diffractometer	2022 independent reflections
Radiation source: Enhance (Mo) X-ray Source	2012 reflections with *I* > 2σ(*I*)
Graphite monochromator	*R*_int_ = 0.026
Detector resolution: 16.0050 pixels mm^-1^	θ_max_ = 25.0°, θ_min_ = 3.6°
Thin slice φ and ω scans	*h* = −7→7
Absorption correction: multi-scan (*CrysAlis RED*; Oxford Diffraction, 2010)	*k* = −8→8
*T*_min_ = 0.851, *T*_max_ = 1.000	*l* = −31→31
15452 measured reflections	

### Refinement

**Table:**

Refinement on *F*^2^	Secondary atom site location: difference Fourier map
Least-squares matrix: full	Hydrogen site location: difference Fourier map
*R*[*F*^2^ > 2σ(*F*^2^)] = 0.018	All H-atom parameters refined
*wR*(*F*^2^) = 0.048	*w* = 1/[σ^2^(*F*_o_^2^) + (0.0259*P*)^2^ + 0.3818*P*] where *P* = (*F*_o_^2^ + 2*F*_c_^2^)/3
*S* = 1.13	(Δ/σ)_max_ = 0.002
2022 reflections	Δρ_max_ = 0.22 e Å^−^^3^
214 parameters	Δρ_min_ = −0.24 e Å^−^^3^
0 restraints	Absolute structure: Flack (1983), 806 Friedel pairs
Primary atom site location: structure-invariant direct methods	Flack parameter: 0.00 (4)

### Special details

**Table:**

Geometry. All s.u.'s (except the s.u. in the dihedral angle between two l.s. planes) are estimated using the full covariance matrix. The cell s.u.'s are taken into account individually in the estimation of s.u.'s in distances, angles and torsion angles; correlations between s.u.'s in cell parameters are only used when they are defined by crystal symmetry. An approximate (isotropic) treatment of cell s.u.'s is used for estimating s.u.'s involving l.s. planes.
Refinement. Refinement of *F*^2^ against ALL reflections. The weighted *R*-factor *wR* and goodness of fit *S* are based on *F*^2^, conventional *R*-factors *R* are based on *F*, with *F* set to zero for negative *F*^2^. The threshold expression of *F*^2^ > 2σ(*F*^2^) is used only for calculating *R*-factors(gt) *etc*. and is not relevant to the choice of reflections for refinement. *R*-factors based on *F*^2^ are statistically about twice as large as those based on *F*, and *R*- factors based on ALL data will be even larger.

### Fractional atomic coordinates and isotropic or equivalent isotropic displacement parameters (Å^2^)

**Table:**

	*x*	*y*	*z*	*U*_iso_*/*U*_eq_	
K	0.43096 (6)	0.06331 (5)	0.592707 (13)	0.01341 (10)	
C1	0.7395 (3)	0.4927 (2)	0.64787 (6)	0.0130 (4)	
C2	0.7781 (3)	0.6005 (2)	0.59853 (6)	0.0101 (3)	
C3	0.9401 (3)	0.7544 (2)	0.61585 (6)	0.0101 (3)	
C4	1.0569 (3)	0.6623 (2)	0.66013 (6)	0.0118 (4)	
H4	1.179 (3)	0.591 (3)	0.6515 (7)	0.014*	
C5	1.0939 (3)	0.8288 (2)	0.69651 (7)	0.0143 (4)	
H5	1.068 (3)	0.790 (3)	0.7284 (8)	0.017*	
C6	0.9307 (3)	0.9816 (3)	0.68001 (7)	0.0163 (4)	
O1	0.5875 (2)	0.39178 (19)	0.65771 (5)	0.0213 (3)	
O2	0.5917 (2)	0.68214 (18)	0.57932 (5)	0.0152 (3)	
O3	1.0843 (2)	0.82586 (17)	0.58160 (5)	0.0132 (3)	
O4	0.8989 (2)	0.52687 (17)	0.68067 (4)	0.0146 (3)	
O5	1.3044 (2)	0.9069 (2)	0.69025 (5)	0.0168 (3)	
O6	0.81153 (19)	0.90096 (17)	0.63873 (4)	0.0133 (3)	
S2	0.89769 (6)	0.42594 (6)	0.555030 (14)	0.01075 (10)	
O21	1.0867 (2)	0.35166 (17)	0.58032 (5)	0.0163 (3)	
O22	0.7275 (2)	0.28416 (18)	0.54814 (4)	0.0170 (3)	
O23	0.9402 (2)	0.53401 (17)	0.50966 (4)	0.0144 (3)	
O8	0.8855 (2)	0.9517 (2)	0.50022 (5)	0.0136 (3)	
O9	0.4146 (3)	0.2464 (2)	0.74597 (5)	0.0243 (3)	
H6A	1.006 (4)	1.106 (3)	0.6699 (7)	0.018 (5)*	
H6B	0.828 (4)	1.008 (3)	0.7059 (8)	0.023 (6)*	
H2O	0.548 (4)	0.623 (4)	0.5572 (9)	0.034 (7)*	
H3O	1.013 (4)	0.860 (4)	0.5587 (9)	0.036 (7)*	
H5O	1.375 (5)	0.847 (4)	0.7033 (10)	0.032 (8)*	
H8OA	0.856 (4)	1.063 (5)	0.5060 (10)	0.049 (9)*	
H8OB	0.775 (4)	0.904 (4)	0.4948 (9)	0.028 (7)*	
H9OA	0.377 (5)	0.146 (5)	0.7374 (11)	0.048 (9)*	
H9OB	0.468 (5)	0.296 (4)	0.7205 (11)	0.042 (8)*	

### Atomic displacement parameters (Å^2^)

**Table:**

	*U*^11^	*U*^22^	*U*^33^	*U*^12^	*U*^13^	*U*^23^
K	0.01291 (17)	0.01296 (18)	0.01436 (18)	−0.00271 (16)	0.00075 (14)	−0.00059 (14)
C1	0.0148 (8)	0.0111 (8)	0.0130 (8)	0.0003 (7)	0.0025 (7)	−0.0026 (7)
C2	0.0099 (7)	0.0094 (8)	0.0111 (8)	0.0005 (7)	0.0002 (6)	−0.0016 (7)
C3	0.0109 (8)	0.0088 (8)	0.0107 (7)	−0.0001 (7)	0.0014 (7)	−0.0007 (6)
C4	0.0121 (9)	0.0111 (8)	0.0121 (8)	−0.0016 (8)	−0.0005 (7)	0.0008 (6)
C5	0.0165 (9)	0.0167 (9)	0.0099 (8)	−0.0046 (8)	−0.0013 (8)	−0.0007 (7)
C6	0.0160 (9)	0.0157 (9)	0.0174 (9)	−0.0009 (8)	−0.0012 (8)	−0.0078 (7)
O1	0.0230 (7)	0.0234 (7)	0.0174 (6)	−0.0114 (7)	0.0046 (6)	0.0005 (5)
O2	0.0115 (6)	0.0157 (6)	0.0183 (6)	0.0036 (6)	−0.0058 (5)	−0.0063 (5)
O3	0.0132 (6)	0.0137 (6)	0.0127 (6)	−0.0036 (6)	−0.0014 (5)	0.0029 (5)
O4	0.0188 (6)	0.0121 (6)	0.0129 (6)	−0.0036 (6)	−0.0004 (5)	0.0021 (4)
O5	0.0132 (6)	0.0164 (7)	0.0208 (7)	−0.0017 (6)	−0.0064 (5)	0.0006 (6)
O6	0.0139 (6)	0.0109 (6)	0.0152 (6)	0.0020 (5)	−0.0032 (5)	−0.0045 (5)
S2	0.0124 (2)	0.00826 (19)	0.01156 (19)	−0.00009 (19)	0.00120 (15)	−0.00172 (16)
O21	0.0160 (6)	0.0148 (6)	0.0183 (6)	0.0044 (6)	−0.0009 (6)	−0.0006 (5)
O22	0.0191 (7)	0.0127 (6)	0.0192 (7)	−0.0032 (6)	0.0028 (5)	−0.0036 (5)
O23	0.0178 (6)	0.0140 (6)	0.0115 (6)	−0.0013 (5)	0.0032 (5)	−0.0011 (5)
O8	0.0131 (7)	0.0118 (7)	0.0158 (6)	−0.0009 (6)	−0.0007 (5)	−0.0020 (5)
O9	0.0335 (8)	0.0240 (8)	0.0154 (7)	−0.0131 (7)	0.0015 (7)	−0.0031 (6)

### Geometric parameters (Å, º)

**Table:**

K—O22	2.6757 (13)	C5—C6	1.531 (3)
K—O3^i^	2.7259 (13)	C5—H5	0.91 (2)
K—O23^ii^	2.8243 (12)	C6—O6	1.443 (2)
K—O2^iii^	2.8466 (13)	C6—H6A	1.02 (2)
K—O6^iii^	2.8934 (12)	C6—H6B	0.96 (2)
K—O5^i^	2.9356 (14)	O2—K^v^	2.8466 (13)
K—O21^iv^	2.9455 (13)	O2—H2O	0.77 (3)
K—O1	3.0265 (13)	O3—K^vi^	2.7259 (13)
C1—O1	1.204 (2)	O3—H3O	0.79 (3)
C1—O4	1.344 (2)	O5—K^vi^	2.9356 (14)
C1—C2	1.537 (2)	O5—H5O	0.70 (3)
C2—O2	1.387 (2)	O6—K^v^	2.8934 (12)
C2—C3	1.538 (2)	S2—O21	1.4495 (13)
C2—S2	1.8364 (17)	S2—O23	1.4517 (12)
C3—O3	1.374 (2)	S2—O22	1.4547 (13)
C3—O6	1.430 (2)	O21—K^vii^	2.9455 (13)
C3—C4	1.529 (2)	O23—K^viii^	2.8243 (12)
C4—O4	1.465 (2)	O8—H8OA	0.81 (3)
C4—C5	1.528 (2)	O8—H8OB	0.78 (3)
C4—H4	0.94 (2)	O9—H9OA	0.77 (3)
C5—O5	1.425 (2)	O9—H9OB	0.83 (3)
			
O22—K—O3^i^	147.16 (4)	O4—C4—C5	110.19 (13)
O22—K—O23^ii^	71.91 (4)	O4—C4—C3	103.95 (13)
O3^i^—K—O23^ii^	76.47 (4)	C5—C4—C3	104.51 (14)
O22—K—O2^iii^	103.48 (4)	O4—C4—H4	107.4 (12)
O3^i^—K—O2^iii^	72.73 (4)	C5—C4—H4	115.7 (12)
O23^ii^—K—O2^iii^	69.45 (4)	C3—C4—H4	114.5 (12)
O22—K—O6^iii^	81.39 (4)	O5—C5—C4	110.47 (15)
O3^i^—K—O6^iii^	117.19 (4)	O5—C5—C6	108.10 (14)
O23^ii^—K—O6^iii^	107.71 (4)	C4—C5—C6	103.82 (14)
O2^iii^—K—O6^iii^	53.58 (4)	O5—C5—H5	112.8 (13)
O22—K—O5^i^	142.42 (4)	C4—C5—H5	110.3 (13)
O3^i^—K—O5^i^	70.30 (4)	C6—C5—H5	110.9 (13)
O23^ii^—K—O5^i^	141.38 (4)	O6—C6—C5	107.03 (13)
O2^iii^—K—O5^i^	82.15 (4)	O6—C6—H6A	111.1 (11)
O6^iii^—K—O5^i^	72.30 (4)	C5—C6—H6A	111.1 (12)
O22—K—O21^iv^	93.52 (4)	O6—C6—H6B	106.8 (13)
O3^i^—K—O21^iv^	79.86 (4)	C5—C6—H6B	111.2 (13)
O23^ii^—K—O21^iv^	93.87 (3)	H6A—C6—H6B	109.5 (18)
O2^iii^—K—O21^iv^	150.46 (4)	C1—O1—K	124.25 (11)
O6^iii^—K—O21^iv^	154.80 (4)	C2—O2—K^v^	128.65 (10)
O5^i^—K—O21^iv^	98.99 (4)	C2—O2—H2O	111.5 (19)
O22—K—O1	66.75 (4)	K^v^—O2—H2O	117.6 (19)
O3^i^—K—O1	140.41 (4)	C3—O3—K^vi^	131.24 (10)
O23^ii^—K—O1	137.02 (4)	C3—O3—H3O	105.2 (19)
O2^iii^—K—O1	131.13 (4)	K^vi^—O3—H3O	110.0 (19)
O6^iii^—K—O1	77.58 (4)	C1—O4—C4	111.14 (13)
O5^i^—K—O1	81.47 (4)	C5—O5—K^vi^	119.38 (10)
O21^iv^—K—O1	77.75 (4)	C5—O5—H5O	107 (2)
O1—C1—O4	122.44 (16)	K^vi^—O5—H5O	120 (2)
O1—C1—C2	126.35 (16)	C3—O6—C6	108.47 (13)
O4—C1—C2	111.20 (14)	C3—O6—K^v^	123.35 (9)
O2—C2—C1	112.80 (14)	C6—O6—K^v^	126.50 (10)
O2—C2—C3	112.01 (14)	O21—S2—O23	115.28 (8)
C1—C2—C3	100.35 (13)	O21—S2—O22	114.05 (8)
O2—C2—S2	111.76 (11)	O23—S2—O22	112.00 (7)
C1—C2—S2	106.78 (11)	O21—S2—C2	105.36 (7)
C3—C2—S2	112.53 (11)	O23—S2—C2	105.37 (7)
O3—C3—O6	113.20 (13)	O22—S2—C2	103.37 (7)
O3—C3—C4	111.05 (14)	S2—O21—K^vii^	151.80 (7)
O6—C3—C4	103.23 (12)	S2—O22—K	146.08 (7)
O3—C3—C2	118.36 (13)	S2—O23—K^viii^	133.30 (7)
O6—C3—C2	104.85 (13)	H8OA—O8—H8OB	104 (3)
C4—C3—C2	104.75 (13)	H9OA—O9—H9OB	105 (3)
			
O1—C1—C2—O2	40.4 (2)	C2—C3—O3—K^vi^	−171.95 (10)
O4—C1—C2—O2	−138.58 (15)	O1—C1—O4—C4	−176.90 (16)
O1—C1—C2—C3	159.75 (17)	C2—C1—O4—C4	2.15 (18)
O4—C1—C2—C3	−19.25 (17)	C5—C4—O4—C1	127.76 (15)
O1—C1—C2—S2	−82.73 (19)	C3—C4—O4—C1	16.26 (17)
O4—C1—C2—S2	98.27 (14)	C4—C5—O5—K^vi^	59.03 (16)
O2—C2—C3—O3	−87.94 (18)	C6—C5—O5—K^vi^	−53.94 (16)
C1—C2—C3—O3	152.15 (14)	O3—C3—O6—C6	−84.25 (16)
S2—C2—C3—O3	38.98 (18)	C4—C3—O6—C6	35.89 (17)
O2—C2—C3—O6	39.38 (16)	C2—C3—O6—C6	145.34 (13)
C1—C2—C3—O6	−80.53 (14)	O3—C3—O6—K^v^	81.84 (15)
S2—C2—C3—O6	166.30 (10)	C4—C3—O6—K^v^	−158.02 (9)
O2—C2—C3—C4	147.71 (14)	C2—C3—O6—K^v^	−48.57 (15)
C1—C2—C3—C4	27.80 (16)	C5—C6—O6—C3	−23.76 (18)
S2—C2—C3—C4	−85.37 (14)	C5—C6—O6—K^v^	170.70 (10)
O3—C3—C4—O4	−156.62 (13)	O2—C2—S2—O21	−179.15 (11)
O6—C3—C4—O4	81.78 (14)	C1—C2—S2—O21	−55.36 (13)
C2—C3—C4—O4	−27.74 (16)	C3—C2—S2—O21	53.81 (13)
O3—C3—C4—C5	87.81 (16)	O2—C2—S2—O23	58.52 (13)
O6—C3—C4—C5	−33.79 (17)	C1—C2—S2—O23	−177.69 (11)
C2—C3—C4—C5	−143.31 (14)	C3—C2—S2—O23	−68.53 (12)
O4—C4—C5—O5	152.70 (14)	O2—C2—S2—O22	−59.16 (13)
C3—C4—C5—O5	−96.18 (16)	C1—C2—S2—O22	64.63 (12)
O4—C4—C5—C6	−91.63 (16)	C3—C2—S2—O22	173.79 (11)
C3—C4—C5—C6	19.50 (18)	O23—S2—O21—K^vii^	−81.35 (17)
O5—C5—C6—O6	118.72 (15)	O22—S2—O21—K^vii^	50.28 (18)
C4—C5—C6—O6	1.37 (18)	C2—S2—O21—K^vii^	162.95 (15)
O4—C1—O1—K	−129.43 (14)	O21—S2—O22—K	67.79 (15)
C2—C1—O1—K	51.7 (2)	O23—S2—O22—K	−159.00 (12)
O22—K—O1—C1	−10.21 (13)	C2—S2—O22—K	−46.05 (15)
O3^i^—K—O1—C1	−166.47 (13)	O3^i^—K—O22—S2	170.05 (11)
O23^ii^—K—O1—C1	−27.09 (16)	O23^ii^—K—O22—S2	−173.73 (14)
O2^iii^—K—O1—C1	77.60 (15)	O2^iii^—K—O22—S2	−110.99 (14)
O6^iii^—K—O1—C1	75.65 (14)	O6^iii^—K—O22—S2	−61.82 (14)
O5^i^—K—O1—C1	149.28 (14)	O5^i^—K—O22—S2	−16.32 (18)
O21^iv^—K—O1—C1	−109.51 (14)	O21^iv^—K—O22—S2	93.35 (14)
C1—C2—O2—K^v^	93.66 (15)	O1—K—O22—S2	18.29 (13)
C3—C2—O2—K^v^	−18.67 (18)	O21—S2—O23—K^viii^	98.09 (10)
S2—C2—O2—K^v^	−146.01 (8)	O22—S2—O23—K^viii^	−34.51 (11)
O6—C3—O3—K^vi^	64.81 (18)	C2—S2—O23—K^viii^	−146.21 (9)
C4—C3—O3—K^vi^	−50.77 (18)		

Symmetry codes: (i) *x*−1, *y*−1, *z*; (ii) *x*−1/2, −*y*+1/2, −*z*+1; (iii) *x*, *y*−1, *z*; (iv) *x*−1, *y*, *z*; (v) *x*, *y*+1, *z*; (vi) *x*+1, *y*+1, *z*; (vii) *x*+1, *y*, *z*; (viii) *x*+1/2, −*y*+1/2, −*z*+1.

### Hydrogen-bond geometry (Å, º)

**Table:**

*D*—H···*A*	*D*—H	H···*A*	*D*···*A*	*D*—H···*A*
O2—H2*O*···O8^ix^	0.77 (3)	1.91 (3)	2.6533 (18)	162 (3)
O3—H3*O*···O8	0.79 (3)	1.87 (3)	2.6525 (18)	171 (3)
O5—H5*O*···O9^x^	0.70 (3)	2.01 (3)	2.684 (2)	162 (3)
O8—H8*OA*···O22^v^	0.81 (3)	2.07 (3)	2.8141 (18)	155 (3)
O8—H8*OB*···O23^ix^	0.78 (3)	2.13 (3)	2.7798 (18)	142 (2)
O9—H9*OA*···O5^i^	0.77 (3)	2.13 (3)	2.869 (2)	161 (3)
O9—H9*OB*···O1	0.83 (3)	1.95 (3)	2.7852 (19)	175 (3)

Symmetry codes: (i) *x*−1, *y*−1, *z*; (v) *x*, *y*+1, *z*; (ix) *x*−1/2, −*y*+3/2, −*z*+1; (x) −*x*+2, *y*+1/2, −*z*+3/2.
